# Hospitals and a Coal Strike

**Published:** 1920-09-18

**Authors:** 


### HOSPITALS AND A COAL STRIKE.

The threatened coal strike would be an ominous prelude to the winter months, and one which would affect hospitals as much as anyone. A coal strike is notably worse in its ultimate effects than a strike of transport workers, because, while hindrances to transport intercept consumption, a coal strike immediately limits supply, and the price of every necessity immediately rises further. The continued advance, even without a coal strike, affects hospitals at a time when they have difficulties enough already, and makes us wonder if the Ministry of Food did not cease its work too soon, since the removal of control always seems to be followed by a rise in prices. Prices have been so much upset because of the war that they have lost all stability; and, if a Ministry of Food was able to accomplish a system of national rationing during the war, it should have regarded a control of food prices afterwards as no less important and surely of no greater difficulty. While, no doubt, the tendency is for everyone to be on the grab at present, we believe that the retailers are particularly unscrupulous, and one of the interesting effects of the Red Cross scheme for buying in bulk and where possible direct from the producer would be to show the extent of the saving, so as to throw some light upon the excessive profits now being made. The price of bread, with the withdrawal of the subsidy, will add an unwelcome increase to institutional bills, and we regret the withdrawal because there is no assurance that the money which has been spent upon the loaf either will relieve taxation or provide the smallest tangible benefit to anybody. The difference between a sixpenny and a shilling loaf is a difference apparent to every man and every institution in the country. The fact that forty-three millions are not being spent on this, so far, tangible result does not offer the smallest guarantee that the sum will be saved or used to pay debts; as likely as not it may merely encourage other expenditure just as costly, but not so easily identified. The immediate effect on the cost of living will add another discouragement to charitable donors, and, since it is extremely unlikely that taxation will be lightened because the bread subsidy has been withdrawn, the discouragement is likely to be permanent.

The effect of a coal strike at the very time when the cost of the loaf will be doubled is likely to be peculiarly disastrous, for the cost of living will be raised in two directions at once, and that, too, at the beginning of the winter. Two additional excuses will be added to the stock list for further demands from the consumer. It is even possible that patients' payments may be more difficult to collect, for how can admission committees in doubtful cases evade the plea that the purchasing power of wages has been much reduced by the shilling loaf and the effects of a coal strike?

With these facts in mind, and the possibility that existing contracts may have to be revised from the above causes, it seems more clear than ever that the system of weekly contributions in London must somehow or other be encouraged. They have, moreover, an advantage peculiar to themselves in that they provide, not merely a permanent source of income which once started freely spreads, but a source of income which the contributors willingly increase. The stable source of hospital revenue to-day is the weekly contribution; and, if certain hospital chairmen criticise the Metropolitan Hospitals Saturday Fund for not doing more, surely it is for them to work to establish weekly contributions in their own locality. This peculiar task, we venture to repeat, has not been tackled seriously. If the coal strike comes and leads to a serious effort to organise these contributions in connection with the London hospitals there will be some set off to its disasters. But hospitals have never had reason to hope for industrial peace more earnestly than at the present time.

